# Assessing the Natural Source Zone Depletion of a Petroleum-Contaminated Clayey Soil Site in Southern China Combining Concentration Gradient Method and Metagenomics

**DOI:** 10.3390/life14030337

**Published:** 2024-03-04

**Authors:** Zhe Xu, Min Zhang, Zhuo Ning, Ze He, Fenge Zhang

**Affiliations:** 1 Institute of Hydrogeology and Environmental Geology, Chinese Academy of Geological Sciences, Shijiazhuang 050061, China; 2120210023@email.cugb.edu.cn (Z.X.); ningzhuozhuo@163.com (Z.N.); heze25@163.com (Z.H.); feng_ezhang@163.com (F.Z.); 2 School of Chinese Academy of Geological Sciences, China University of Geosciences, Beijing 100083, China; 3 Key Laboratory of Groundwater Remediation of Hebei Province & China Geological Survey, Shijiazhuang 050061, China; 4 Comprehensive Survey and Management Center for Natural Resources, China Geological Survey, Beijing 100055, China

**Keywords:** natural source zone depletion (NSZD), LNAPL removal, concentration gradient method, clayey soil, metagenome, methanogenic functional gene

## Abstract

Natural source zone depletion (NSZD) is the main process of LNAPL (Light Non-Aqueous Phase Liquid) removal under natural conditions. The NSZD rates assessed ranged from 0.55 to 11.55 kg·m^−2^·a^−1^ (kilograms per square meter per year) in previous studies. However, most of these data were obtained from sandy sites, with few clayey sites. To gain knowledge of NSZD in clayey soil sites, the study assessed the NSZD of a petroleum hydrocarbon-contaminated clayey soil site in China, combining the concentration gradient method with metagenomic sequencing technology. The results show that the abundance of methane-producing key enzyme *mcr*A gene in the source zone was more abundant than in background areas, which suggests that there was methanogenesis, the key process of NSZD. The concentration gradients of oxygen and carbon dioxide existed only in shallow soil (<0.7 m), which suggests that there was a thin methane oxidation zone in the shallow zone. The calculated NSZD rates range from 0.23 to 1.15 kg·m^−2^·a^−1^, which fall within the moderate range compared to previous NSZD sites. This study expands the knowledge of NSZD in clayey soil and enriches the attenuation rate data for contaminated sites, which is of significant importance in managing petroleum contaminants.

## 1. Introduction

Natural Source Zone Depletion (NSZD) refers to the process in which LNAPL (Light Non-Aqueous Phase Liquid) in the source zone (the area where mobile and residual contaminants exist) is removed through natural attenuation mechanisms such as volatilization, dissolution, and biodegradation [1,2,3]. In the case of small-volume LNAPL spills, the LNAPL may be trapped in the soil pores of the vadose zone. However, for sufficient LNAPL spills, an immobile residual phase may form along the migration pathway, accumulating primarily at the groundwater table [4]. Then, LNAPL spreads along the direction of groundwater flow. Current studies indicate that biodegradation of LNAPL in the saturated zone and the vadose zone are the two fundamental processes contributing to NSZD, with the natural attenuation in the vadose zone accounting for 90% to 99% of the total mass loss of LNAPL [5,6,7]. Based on the key processes of NSZD, a one-dimensional conceptual model was proposed that divides the NSZD zone above the LNAPL body into three zones: A methane generation zone, methane oxidation zone, and aerobic transport zone [8]. Significant methanogenesis occurs at the saturated zone and the bottom of the vadose zone, which results in generating methane (CH_4_) and carbon dioxide (CO_2_) bubbles that migrate vertically upward through the vadose zone [9]. There may also be direct outgassing of LNAPL occurring at the bottom of the unsaturated zone. The combined gas fluxes migrate vertically upward through the vadose zone to a relatively thin reaction zone where most or all of the methane and volatile organic compounds (VOCs) are oxidized by methane-oxidizing bacteria, consuming oxygen (O_2_) and producing carbon dioxide. Then, in the shallow soil, the generated carbon dioxide and any remaining unoxidized hydrocarbons continue to diffuse upward, and atmospheric oxygen diffuses toward the methane oxidation zone [1,8].

The methods currently used to assess NSZD rates of petroleum hydrocarbon-contaminated sites mainly rely on subsurface gas fluxes and thermal fluxes associated with natural degradation processes [10]. Commonly used methods include the gas concentration gradient method, dynamic chamber method, carbon capture method, and thermodynamic gradient method [10,11,12]. Many studies have used different methods to evaluate the natural attenuation of contaminated sites, even making improvements and corrections to these methods [10,13,14,15,16,17,18]. Among them, the concentration gradient method was the earliest proposed, and it is currently the most mature and commonly used method, as the data required for the concentration gradient method is the easiest and fastest to obtain on-site [1]. Different depth soil gas samples are collected along the vertical soil profile to measure the concentrations of various components (O_2_, CO_2_, CH_4_, VOCs) in the soil gas, which are used to estimate the LNAPL source zone depletion (NSZD) rate. By combining the profile soil gas concentrations, the reaction stoichiometry of organic compounds during degradation, and the effective diffusion coefficient in Fick’s first law, the depletion rate of underground LNAPL can be estimated [1,19,20]. Factors that can cause errors in the rates obtained from the gradient method include correction for natural soil respiration, barometric pumping, surface wind, precipitation and/or soil moisture, artificial surfaces, and heterogeneities in diffusion coefficients [10]. Since the conceptual model of NSZD was proposed, along with the application of the concentration gradient method in the assessment of contaminated sites, an increasing number of studies have begun to adopt the concentration gradient method to evaluate the natural attenuation of contaminated sites [21,22].

Using methods such as concentration gradient, carbon capture, and thermodynamic gradient, NSZD rates ranging from 0.05 to 11.55 kg·m^−2^·a^−1^ (kilograms per square meter per year) have been obtained for dozens of petroleum-contaminated sites in previous studies [5]. However, most sites are characterized by sandy soils, with few assessments conducted on other types of formations, particularly the clay layer, which is widely distributed in various regions of China and other areas such as France and the United States, etc. The abundance of clay minerals and humic matter in the clayey soil contributes to providing more nutrients for microorganisms, changing their metabolism and promoting their growth [23]. Its high surface area and cation exchange capacity may provide better access for microorganisms to the matter accumulated on the clay surfaces [24,25]. Therefore, it is likely to be more conducive to the removal of hydrocarbons/methane, resulting in faster degradation rates. On the other hand, the low permeability of the clay layer may impede the diffusion and escape of gases. Therefore, it is hard to say whether the NSZD rate in the clay-soil is faster or slower than that in the sand-soil studied in the past, or whether there is no difference between the two. Additionally, in many site assessment studies, there is insufficient evidence regarding the presence of microbial processes involved in clay or other geological formations [1,5,26].

Thus, aiming at these problems, this study assessed the NSZD rate of a clayey soil site contaminated with LNAPL in China using the gas concentration gradient method and determined the occurrence range of NSZD processes in the vadose zone combining metagenomic sequencing technology. This study is expected to expand the insight of NSZD in clayey petroleum-contaminated sites and enrich the attenuation rate data for contaminated sites.

## 2. Materials and Methods

### 2.1. Site Description

**Figure 1:**
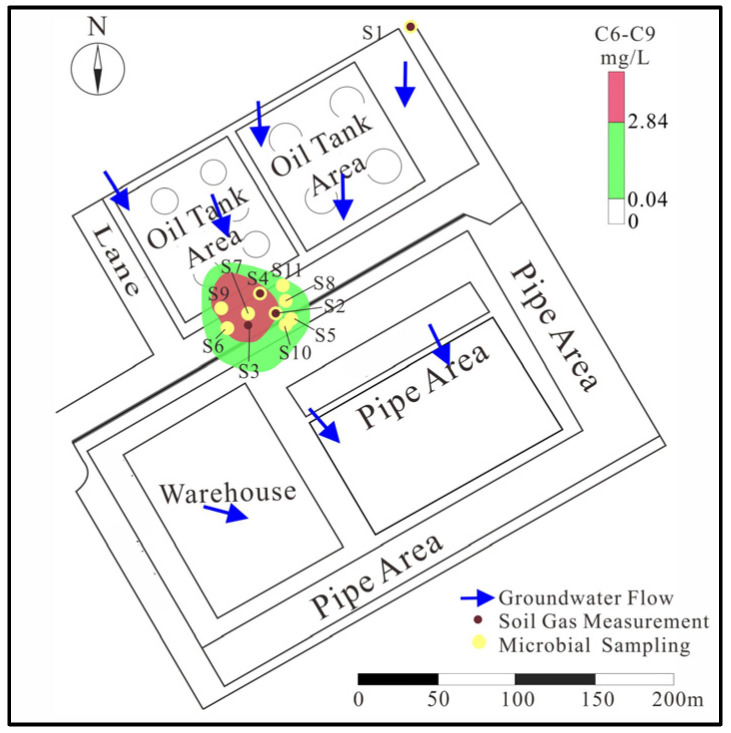
The characteristics of the study area and LNAPL contaminant. The plot shows the contaminant source (red area), contaminant plume (green area), groundwater flow direction, soil measurement points, and microbial sampling points.

The study area is located near an oil tank area of a petrochemical site in the Anhui Province, China. The vadose zone is primarily composed of silty clay and clay, which are clayey soil, from top to bottom. The thickness of the vadose zone is less than 3 m. The groundwater flow direction is from north to south, roughly (Figure 1). The contaminants are mainly light petroleum hydrocarbons from oil tank leakage, such as benzene and other small molecule volatile petroleum hydrocarbons (C_6_–C_9_). Under the impact of the groundwater flow, the source zone is located in the southern part of the tank area. Vertically, the heavily contaminated zone is mainly found at depths of 2–4 m (within the fluctuation zone of the water table). Some localized contamination exists in the upper part of the heavily contaminated zone. The contamination at the site displays significant heterogeneity both horizontally and vertically due to spilling during the transportation and the difference in the migration ability of contaminants.

According to the distribution of contaminants in the site, five points, S2, S4, S6, S7, and S9, at the contaminant source zone, and four points, S5, S8, S10, and S11, at the contaminant plume zone, were selected to determine metagenome information. Due to the site conditions, three points in the source zone were selected at random as soil gas sampling locations (S2–S4). Meanwhile, one point (S1) on the north side of the tank area, the upstream source area, was selected as the background (Figure 1).

### 2.2. Soil Gas Measurement and Functional Gene Determination

#### 2.2.1. Soil Gas Measurement

**Figure 2:**
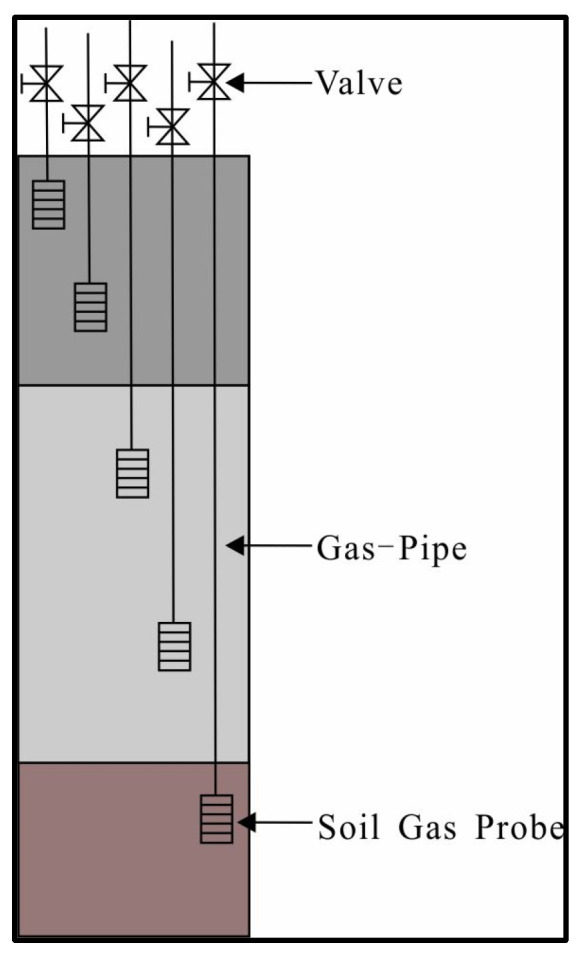
The schematic of the gas monitoring well. The different colors in the figure are used only to indicate that the monitoring well may cover soil layers with different characteristics, and do not represent the actual formation condition of the site in this study.

In all instances, measurements should be conducted above the LNAPL footprint. However, the specific depth and width of these measurements may vary depending on the distribution of LNAPL and vapor [27]. Limited by tank area location, it was not possible to deploy wells for gas measurement in the area. Since the heavy contaminant mainly occurs at a depth of 2–4 m, the measurement was made at a depth of 0–2.0 m. Referring to the “Technical Guideline of Soil and Groundwater Self-Monitoring for Enterprises in Production”, 5.0 cm-diameter and 2 m-depth boreholes were drilled at the selected points as gas monitoring wells [28]. Multi-level soil gas probes were buried (0.1 m intervals). Portable multi-parameter gas detectors were used to measure the concentrations of gases, such as O_2_, CH_4_, CO_2_, and VOCs, in the soil through gas conduits. Data was read every 30 s during the measurement. For gas indicators whose trend of data change was only an increase or a decrease, three consecutive readings with no change were considered the stable state. Figure 2 shows the schematic of the gas monitoring wells. Due to the limited soil gas that can be extracted from the monitoring well to reflect the real situation, it was not appropriate to extract gas for a long time, or a large amount of gas in a short time, through monitoring wells. Therefore, as long as it was guaranteed that the gas in the soil had been extracted by the detector during measurement to reach a stable state, the obtained data from a single measurement would be representative. This monitoring method allows for the cost-effective acquisition of concentration data directly from different layers in a short time. Compared to a minimally invasive measurement, this method provides more intuitive and accurate soil gas data [1,29].

#### 2.2.2. Functional Gene Determination

In this study, groundwater was extracted from wells, and DNA was collected to detect the functional gene mcrA. Before water sampling, the wells were washed until the physics and chemistry parameters of the groundwater were stable. After the well-washing process, representative groundwater samples were collected and transferred to 5 L sterile plastic containers. Samples were protected from light and refrigerated for transportation to the laboratory for DNA extraction. The DNA was then collected by a vacuum pump (SHB-III A) onto filter membranes with a pore size of 0.22 μm. After the collected DNA was extracted, fragmented, and sequenced on the Illumina HiSeq4000 platform (Illumina Inc., San Diego, CA, USA) at Majorbio Bio-Pharm Technology Co., Ltd., (Shanghai, China) data quality control was performed [30]. The gene prediction was carried out after assembling the short sequences obtained from the quality control. Then, the non-redundant gene set was constructed. Finally, a KEGG annotation was performed. The non-redundant gene set sequences were compared with KEGG’s gene database (GENES) using BLASTP. Here, ppm was used to calculate the abundance of functional genes, i.e., the number of reads corresponding to a gene per million sequenced reads [30]. The specific method for functional gene determination is the same as the previous metagenomic testing techniques using conventional experimental techniques [31,32,33].

### 2.3. Assessment Method

According to the definition of “source zone” and the leakage situation of LNAPL, LNAPL is typically present in both the vadose zone and the saturated zone. A portion of contaminants will dissolve into the groundwater and form a contaminant plume that migrates downstream, while another portion will volatilize upward through the vadose zone [34,35]. During the physical processes of dissolution and volatilization, significant biodegradation occurs at the bottom of the saturated zone and vadose zone due to the activity of methanogenic bacteria. Contaminant components and their degradation products volatilize and diffuse away from the source zone, while oxygen required for aerobic biodegradation diffuses downward from the atmosphere. The mass loss rate of LNAPL related to these processes is calculated through the vertical vapor flux, which is located on a horizontal plane above the source zone in the vadose zone [17]. The following assumptions are made: the oxygen flux is caused by a combination of direct aerobic biodegradation of the source, aerobic biodegradation of vapor in the source zone, and aerobic biodegradation of methane (if any) produced by anaerobic degradation/methanogenesis in the source zone; the background oxygen utilization rate and background methane production below the ground can be neglected; diffusion is the main controlling mechanism for gas flux, and the vadose zone soil is homogeneous and isotropic. Based on these assumptions, the mass loss rate of NSZD related to vapor transport processes is calculated [1,36].

After obtaining the concentrations of VOCs, CH_4_, and O_2_ from one measurement at every point, the NSZD rate of the site was evaluated using Formula (1) [1,36]:

(1) $$R=-D_{\mathrm{eff},HC}\frac{\partial C_{HC}(x)}{\partial x}-S_{CH_{4}}\cdot D_{\mathrm{eff},CH_{4}}\frac{\partial C_{CH_{4}}(x)}{\partial x}+S_{O_{2}}\cdot D_{\mathrm{eff},O_{2}}\frac{\partial C_{O_{2}}(x)}{\partial x}$$

where *R* is the rate of NSZD mass loss associated with the vapor transfer process (kg·m^−2^·a^−1^); $D_{eff,HC}$, $D_{eff,CH_{4}}$, and $D_{eff,O_{2}}$ are the effective diffusion coefficients for hydrocarbons (represented as HC), CH_4_, and O_2_ within the range of measuring gas concentrations (m^2^/d); $\frac{\partial C_{HC}\left(x\right)}{\partial x}$, $\frac{\partial C_{CH_{4}}\left(x\right)}{\partial x}$, $\frac{\partial C_{O_{2}}\left(x\right)}{\partial x}$ are vertical concentration gradients of HC, CH_4_ and O_2_ (kg/L/m); and $S_{CH_{4}}$ and $S_{O_{2}}$ are stoichiometric numbers of anaerobic methanogenesis reactions of hydrocarbons and aerobic oxidation of hydrocarbons and methane (kg HC/kg CH_4_ and kg HC/kg O_2_)

The effective diffusion coefficient of gas is estimated by Formula (2) [37,38]:

*D_eff_* = *D_air_*·*θ_gas_*^3.3^_/_*θ_total_*^2^ (2)

where *D_eff_* represents the effective diffusion coefficients for hydrocarbons, CH_4_, and O_2_ within the range of measuring gas concentrations (m^2^/d); *D_air_* is the diffusion coefficients for hydrocarbons, CH_4_, and O_2_ in air (m^2^/d); *θ_gas_* is the volume proportion of gas in the porosity of the vadose zone; and *θ_total_* is the porosity of the vadose zone.

Underground microorganisms can utilize petroleum hydrocarbons as carbon and energy sources for growth [39]. In the subsurface environment, contaminants can undergo denitrification, iron reduction, sulfate reduction, and methane production reactions. Each of these different reaction processes corresponds to different microbial populations and associated functional genes. In the process of natural source zone depletion, methane production, and methane oxidation are two key biological processes [8]. The higher the abundance of microorganisms and functional genes associated with methane production and methane oxidation, the stronger the reaction degree of the process, and the greater the contribution to the degradation of hydrocarbons/methanogenesis. If these processes do not occur, the abundance will not be high. The samples obtained in this study are from the water table fluctuation zone, and the microbial results in them can reflect the methane production in the saturated zone and the bottom of the vadose zone. The McrA gene is a key gene in the last step of methanogenesis metabolism [40]; therefore, the presence of functional genes (mcrA) associated with the degradation of characteristic contaminants can serve as evidence of methane production and natural attenuation in the source area [41].

### 2.4. Statistical Calculation

The statistical analysis was made in the statistical packages of Origin 2018 (the version number, 9.5.0.193) and Microsoft Excel 2016 (the version number, 16.0.17328.20124). Point plots and a bar graph were obtained from Origin 2018. Point plots displayed the distribution and changes of soil gases. The bar graph showed the abundance of the mcrA gene at each point, as well as the differences between points. The linear fittings of data were completed by Microsoft Excel 2016. R^2^ greater than 0.70 is considered to be a good fit.

## 3. Results

### 3.1. Soil Gas Concentration Profile

**Figure 3:**
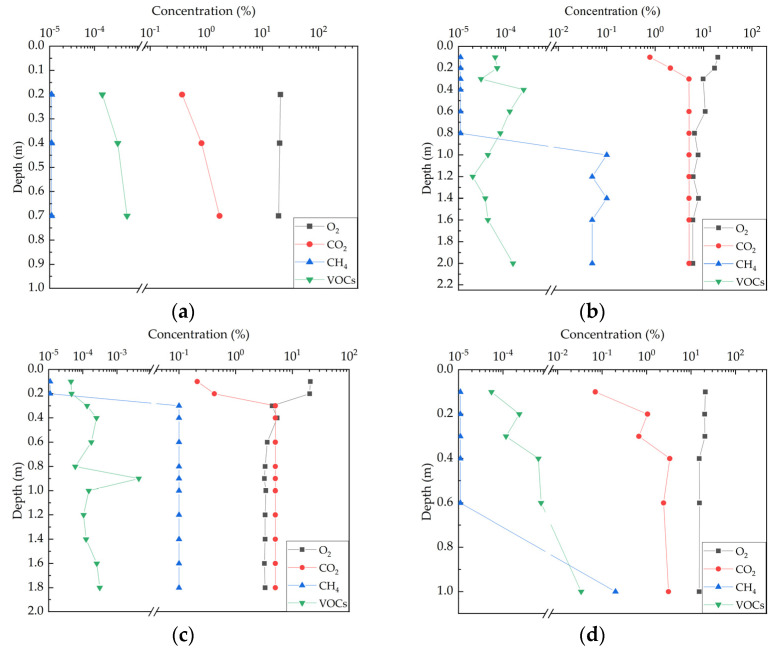
Soil gas profile concentration distribution. Plot (**a**) represents the soil gas concentration of S1 point; Plot (**b**) represents the soil gas concentration of S2 point; Plot (**c**) represents the soil gas concentration of S3 point; Plot (**d**) represents the soil gas concentration of S4 point.

Based on the obtained soil gas data, the vertical concentration profiles of four types of gases, O_2_, CO_2_, CH_4_, and VOCs, at different depths were determined in Figure 3:

O_2_, CO_2_, and CH_4_ exhibit distinct segmented characteristics, but the depth at which the profiles of several points are “divided into two segments” is not consistent. For point S2, between 0 and 0.3 m depth, soil oxygen concentration decreases rapidly (linear fit R^2^ = 0.94), carbon dioxide increases rapidly (linear fit R^2^ = 0.95), and methane is not detected; below 0.3 m depth, oxygen concentration decreases overall, shows fluctuating changes below 0.6 m depth, methane concentration increases between 0.3 and 1.0 m (linear fit R^2^ = 0.91), and then fluctuates. The carbon dioxide concentration reaches 5% and no longer changes. This is because the CO_2_ concentration exceeds the upper detection limit of 5% concentrate value due to the limitations of the on-site testing instrument. Theoretically, it should be opposite to the trend of oxygen changes (the carbon dioxide concentration at point S3 also exhibits this situation, similarly). For point S3, between 0 and 0.3 m depth, soil oxygen concentration decreases rapidly (linear fit R^2^ = 0.77), carbon dioxide increases rapidly (linear fit R^2^ = 0.78), and methane concentration increases (linear fit R^2^ = 0.75); below 0.3 m depth, oxygen concentration decreases overall, shows fluctuating changes below 0.8 m depth, methane concentration remains unchanged, and carbon dioxide concentration reaches 5%. For point S4, between 0 and 0.4 m depth, soil oxygen concentration decreases rapidly (linear fit R^2^ = 0.94), carbon dioxide increases rapidly (linear fit R^2^ = 0.95), and methane is not detected; below 0.4 m depth, oxygen, and carbon dioxide concentrations show fluctuating changes, no longer changing linearly, and methane concentration increases (linear fit R^2^ = 0.99).

From the above Figure 3a–d, it can be observed that all soil gas profiles show consumption of oxygen and production of carbon dioxide. Oxygen, carbon dioxide, and methane exhibit distinct two-phase patterns: in the shallow soil, as depth increases, oxygen concentration decreases rapidly, carbon dioxide increases rapidly, and methane is almost not detected; below a certain depth, the rate of change in oxygen and carbon dioxide slows down, showing some fluctuations, and methane is detected. This is consistent with the hypothesis of a combination of aerobic and anaerobic biodegradation in the source zone [1,36]. Under the conditions of vertical one-dimensional gas transport, NSZD leads to a decrease in oxygen concentration and an increase in carbon dioxide concentration increases with depth. Oxygen concentration and carbon dioxide concentration are negatively correlated. Methane concentration in the lower part of the vadose zone is higher than in the shallow soil. The oxygen concentration at background point S1 is higher than in the source zone, and the carbon dioxide and methane concentrations are lower than in the source zone.

It can be clearly observed that VOCs are present at different depths at different points, and their concentrations do not vary regularly with depth. They reach maximum values at 0.4 m (S2), 0.9 m (S3), and 1.0 m (S4), which are two–three orders of magnitude higher than other layers. Combined with the soil contaminant situation in the site investigation, beyond the presence of a large amount of contaminant in the heavily contaminated area, there is also an isolated and uneven small-scale point-like petroleum hydrocarbon contaminant in the upper part. Therefore, the VOCs detected in the soil are most likely due to the volatilization of shallow to intermediate depth contaminants. The closer it is to these positions, the higher the VOC concentration. The contaminants diffuse upward and downward from this position, resulting in the highest VOCs concentration at intermediate depths. Comparing the concentrations of volatile gases and oxygen at each point, it is found that the concentration of volatile gases is lower. Compared with other processes, the mass loss caused by direct volatilization from the source zone is small. It is more likely that the mass loss is due to the aerobic biodegradation processes related to the downward diffusion of oxygen through the vadose zone.

### 3.2. Soil Gas Concentrate Gradient

The application of the concentration gradient method, as mentioned earlier, is based on several key assumptions. Previous studies have indicated that advective flux only occurs in the adjacent LNAPL source zones, while diffusion serves as the primary gas transport mechanism in the upper aerobic zone [27]. In this study, the locations for obtaining soil gas concentration include the aerobic zone above the soil affected by hydrocarbon compounds. Additionally, in practical applications, underground soil is rarely homogeneous and isotropic. Although this may introduce uncertainty, it does not affect the use of the gradient method. It is usually not necessary to obtain the gas effective diffusion coefficient for the entire vadose zone, but only within the depth range of soil gas measurements. The overall gas effective diffusion coefficient can be calculated by weighting the gas diffusion coefficients obtained from different permeable layers [1,36]. Preliminary site investigations and data indicate that the permeability, porosity, and water content near the soil tank area are similar. The gas effective diffusion coefficient is a function of porosity and water content, so similar porosity and water content result in consistent gas effective diffusion coefficients [42]. Taken together, the soil gas concentration gradient method is applicable for NSZD assessment at this site.

For points S2 and S4, since the methane concentration of the boundaries where the gas profiles are divided into two stages is exactly zero, for ease of calculation, the NSZD rates are calculated using the oxygen flux from 0 to 0.3 m and 0 to 0.4 m at these two points. However, point S3 does not meet this condition. If the calculation plane is set at a depth where methane concentration is zero, then the concentration gradient of oxygen is significantly smaller, so, the calculated NSZD rate will be greatly underestimated. Therefore, the NSZD rate is calculated using the oxygen and methane flux from 0 to 0.3 m at point S3. The VOC concentrations measured in the soil are three–five orders of magnitude lower than the CO_2_ concentration, and the gas flux is very low. Therefore, the LNAPL mass loss caused by source volatilization is neglected in the calculations.

Table 1 calculated the concentration gradients at each sampling point based on the gas concentrations at different depths. For points S1, S2, S3, and S4, the O_2_ concentration gradient was −4.29 × 10^−5^, −7.00 × 10^−5^, −1.16 × 10^−5^, and −2.67 × 10^−5^ kg/L/m. The CO_2_ concentration gradient was 5.30 × 10^−5^, 4.13 × 10^−5^, 4.70 × 10^−5^, and 2.13 × 10^−5^ kg/L/m, respectively. The CH_4_ concentration gradient for S3 was 0.5 kg/L/m, and it was 0 for other points. Respectively, the VOCs concentration gradient was 2.65 × 10^−8^, 5.22 × 10^−8^, 1.53 × 10^−8^, and 6.60 × 10^−8^ kg/L/m.

### 3.3. Assessment of NSZD Rate

According to Johnson [1], it is difficult to accurately determine the stoichiometric coefficient S_O2_ for aerobic biodegradation of hydrocarbons without knowing their composition. The range is approximately 0.25 to 0.29 kg-HC/mg-O_2_, depending on the relative contributions of direct aerobic oxidation (0.29 kg-HC/mg-O_2_) and indirect oxidation (0.25 mg-HC/mg-O_2_). Here, we use 0.29 kg-HC/mg-O_2_.

Based on Formula (2), for calculating the effective diffusion coefficients of gases, the effective diffusion coefficients for O_2_, CO_2_, and CH_4_ in the depth range of measuring gas concentration are 9.53 × 10^−3^ m^2^/d, 7.23 × 10^−3^ m^2^/d, and 1.02 × 10^−2^ m^2^/d, respectively [29].

At sampling points S2, S3, S4, and the background point S1, the organic compound attenuation rates caused by methane volatilization and aerobic oxidation of hydrocarbons and methane are 0.71 kg·m^−2^·a^−1^, 1.19 kg·m^−2^·a^−1^, 0.27 kg·m^−2^·a^−1^, and 0.04 kg·m^−2^·a^−1^, respectively (Table 2). In Table 2, the calculated rate represents the NSZD rate calculated using formula (1). Correction for calculated rate represents the NSZD rate minus the background value. After subtracting the background, the site attenuation rate ranges from 0.23 kg·m^−2^·a^−1^ to 1.15 kg·m^−2^·a^−1^, which falls between 25% (0.20 kg·m^−2^·a^−1^) and 75% (1.93 kg·m^−2^·a^−1^) of dozens of historical sites attenuation rates [5], indicating a moderate level of attenuation.

When using the concentration gradient method for calculation, the volatilization flux of VOCs is ignored, and the attenuation of LNAPL is assumed to be caused by aerobic oxidation of source VOCs and CH_4_ during volatilization. However, if the flux of degradation product CO_2_ is used for calculation, the attenuation rates are 0.33 kg·m^−2^·a^−1^, 0.37 kg·m^−2^·a^−1^, 0.17 kg·m^−2^·a^−1^, and 0.04 kg·m^−2^·a^−1^, respectively. The attenuation rate range obtained using the carbon dioxide flux method will be 0.13 kg·m^−2^·a^−1^ to 0.33 kg·m^−2^·a^−1^.

### 3.4. Functional Genes Evidence

**Figure 4:**
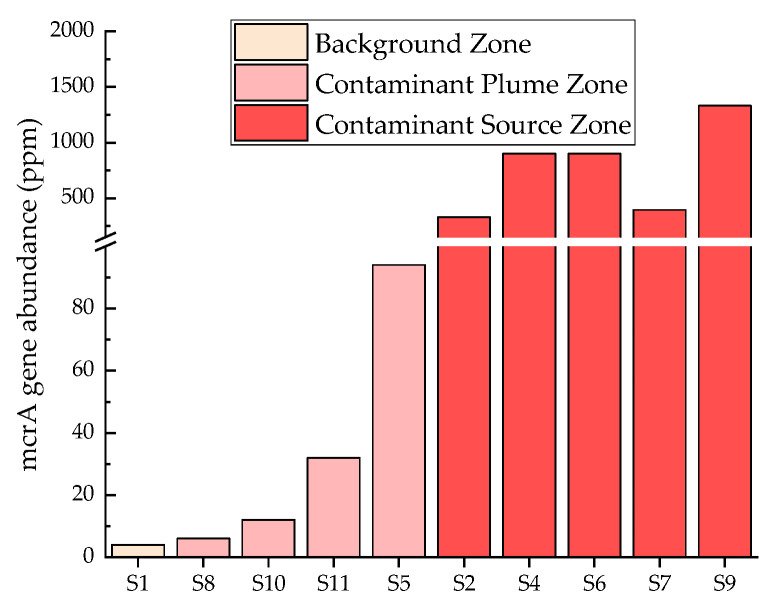
McrA gene abundance.

In the 10 sampling points, the methane-producing key enzyme mcrA gene was detected at varying levels (Figure 4). The gene abundance ranged from 4 ppm to 1332 ppm. In Figure 4, it was obvious that the mcrA gene abundances in the contaminant source zone were greater than those in the contaminant plume zone. Both were far greater than the gene abundance in the background zone. The functional gene abundance of the background point S1 is 4 ppm, indicating a relatively low level. Among the samples near the oil tank area, the mcrA gene abundance at point S9 is the highest, reaching 1332 ppm. The lowest is 6 ppm in point S8, which is close to the background point. Samples with high gene abundance are mainly located in the source area. Among the two points S2 and S4, S4 had a relatively high gene abundance.

## 4. Discussion

The concentration gradient of soil gases indicates that, as the O_2_ concentration decreases, the CO_2_ concentration increases. It is suggested that there is the presence of O_2_ consumption in the lower part accompanied by the production of CO_2_. The concentration of VOCs is influenced by both the volatilization of LNAPL in the lower vadose zone and the contamination of shallow petroleum hydrocarbons. However, the concentration of VOCs is still three–five orders of magnitude lower than that of CO_2_, indicating that only a small fraction of VOCs volatilize to the surface in the source area. During the volatilization process, there is also biological degradation, resulting in low VOC concentrations, which is consistent with the current understanding of the conceptual model of natural source zone depletion [3,12]. The O_2_ concentration at the background point S1 shows very little O_2_ consumption within the vadose zone. It is higher than the source area, and the CO_2_ concentration is lower than the source area. There is a lower concentration gradient of oxygen and carbon dioxide in the background soil than the source area points. The significant O_2_ consumption in the source area at a short distance (that means a large concentration gradient) from the ground surface indicates a high natural source zone depletion rate at that location [27]. The calculated rate of 0.04 kg·m^−2^·a^−1^ using the gradient method may reflect the soil respiration rate more. The calculated attenuation rates at other points are greatly higher than the background point, indicating the presence of natural attenuation in the source area at points S2, S3, and S4.

According to a large number of field case data [1,3,5,11,12,20,29,36], there are generally two situations in the profile gas concentration. One is that, within a considerable depth range from the surface to the contaminated source area, oxygen and carbon dioxide have a constant concentration gradient, and methane is almost non-existent within this range. The other situation is that, within a considerable depth range, when the oxygen concentration decreases to a certain value and the carbon dioxide concentration increases to a certain value, the concentration gradient of the two gases begins to decrease, and the methane concentration increases from top to bottom. Elevated CH_4_, with an absence of O_2_, is an indicator that a location is below the aerobic oxidation zone [43]. When methane and oxygen meet, a relatively thin oxidation zone is formed, and methane and volatile hydrocarbon (if any) are converted to carbon dioxide [8]. The position of the oxidation zone is influenced by the downward migration of O_2_ through the surface and the impact range of subsurface hydrocarbons. The methane oxidation rate within the zone is limited by the diffusion rate of oxygen in the atmosphere [43]. It is evident that this study falls into the latter between the two situations, with a sudden change in concentration gradient near a depth of 0.3–0.4 m. It can be inferred that there is a relatively narrow aerobic oxidation zone near a depth of 0.3–0.4 m, with an aerobic transport zone above and a methane production zone below. The oxygen gradient is smaller in the methane production zone. Among the previous studies, there were few sites where aerobic oxidation zones existing at this depth. Different from these sites, which are characterized by sandy soils, this site is characterized by clay [5,44,45].

The position of the aerobic oxidation zone for methane and hydrocarbons is influenced by multiple factors, such as recent releases (early stage), less soil permeability in the upper portion of the vadose zone, high methane production rate in the subsurface, and extensive low permeability surface cover, which all allow oxidation zones to form in shallow layers [43]. Otherwise, the oxidation zone will move downward. The clayey layer’s poor permeability at the study site is likely the main reason for the shallow oxidation zone, as it it limits the downward diffusion of oxygen.

Methane gas can be detected in the vadose zone, directly indicating the presence of methane production in the saturated zone underground or vadose zone. There are three pathways to produce methane. Methanogens use H_2_, methyl compounds, or acetates as sources for growth and methanogenesis [46,47]. The key functional gene in methane production is methyl-coenzyme M reductase (mcrA), which is responsible for the final step of methane production in methanogenesis and the activation reaction in methane oxidation metabolism [48,49]. It exists no matter how methane is produced. The high abundance of mcrA near the oil tank area and detection of methane gas in groundwater at corresponding points (Figure S1) indicate the presence of methane production in the fluctuation zone of the water table, again. The points with high gene abundance, such as S9, S4, and S6, have the greatest potential for methane degradation. Among the three points S1, S2, and S4, S4 has the highest abundance of mcrA genes and the greatest potential for methane production, while the background point S1 has the lowest gene abundance and the smallest potential for methane production. The methane gas concentration in the vadose zone at points S2 and S4 is consistent with the gene abundance. The enrichment culture can be obtained in the laboratory to further verify the current methanogenic activity of the site.

The attenuation rates calculated using the oxygen concentration gradient range from 0.23 kg·m^−2^·a^−1^ to 1.15 kg·m^−2^·a^−1^, while the attenuation rates calculated using the carbon dioxide concentration gradient range from 0.13 kg·m^−2^·a^−1^ to 0.33 kg·m^−2^·a^−1^. It can be observed that the attenuation rates calculated using the carbon dioxide flux are lower than those calculated using the oxygen flux, but the relative magnitudes of the results obtained from both methods are consistent. This may be due to limitations in the instrument detection, as the maximum detection limit for carbon dioxide is 5%, which may not reflect the truest results. For background and low-rate locations S4, the carbon dioxide test values did not reach the detection limit, and the results from both methods were similar. Additionally, for the low-rate location S4, the result obtained from the carbon dioxide concentration gradient calculation was slightly lower than that obtained from the oxygen concentration gradient calculation. This may be explained by the fact that CO_2_ is the final product of the biodegradation of hydrocarbons, and during the biodegradation process, some hydrocarbons may be converted into other intermediate products before being converted into CO_2_. These intermediate products were not considered in the estimation of NSZD using CO_2_ flux. However, when using O_2_, hydrocarbons and O_2_ have a corresponding relationship (stoichiometry), so even if some hydrocarbons have not been completely biodegraded into CO_2_, their consumption of O_2_ will be included in the NSZD calculation. This may result in a lower attenuation rate estimated using the CO_2_ flux. Therefore, in the assessment of natural attenuation in source zones, if a significant number of hydrocarbons is converted into intermediate products instead of CO_2_, the attenuation rate estimated using the CO_2_ flux method may be significantly underestimated, which is similar to previous studies [29].

Some studies have suggested that the distribution of O_2_ and CO_2_ concentrations in some LNAPL source zones is not linear but exhibits a curved or semi-curved pattern. Assuming a linear distribution of concentrations may underestimate the concentration gradient [45]. Based on a first-order reaction model, a new method was proposed for calculating concentration gradients. In the method, the maximum value of oxygen O_2, max_ divided by the reaction length LR_O2_ was used to calculate the concentration gradient, and then the gas flux was calculated based on Fick’s first law. According to the field data of this study, using this method, the attenuation rates at points S2 to S4 were calculated to be 0.27 kg·m^−2^·a^−1^, 0.56 kg·m^−2^·a^−1^, and 0.06 kg·m^−2^·a^−1^, respectively, with a range of 0.06 kg·m^−2^·a^−1^ to 0.56 kg·m^−2^·a^−1^. The results obtained from this method are approximately half of the results obtained from this study. The difference is within an acceptable limit. It is proven that the linear hypothesis of concentration is feasible to apply in this site.

Comparing the results of several dozen previous site assessments, the rates obtained in this assessment are at a medium to high level among these results. It is found that although there are significant differences in the soil conditions of the vadose zone, the results of this assessment are similar to the range of results from other sites [8]. This indicates that although the clayey soil of the vadose zone significantly affects the position of the aerobic oxidation zone, it does not significantly increase the attenuation rate. The degradation rate in the site is influenced by multiple factors. The moisture content in clayey soil is higher than in sandy soil, resulting in a smaller effective diffusion coefficient, according to Formula (2). When the gas concentration gradient is equal to it in sandy soil, the smaller effective diffusion coefficient in clayey soil may lead to a lower depletion rate because of the reduced gas flux. In fact, the gas concentration gradient in this clayey soil field is larger than that in a sandy soil field with the same depletion rate. The larger concentration gradient indicates that there is probably more active microbial activity below the surface. Although studies have also shown that the complexes of clay and humic acids stabilize complex organic compounds, such as highly labile biopolymers like proteins, polysaccharides, DNA, etc., resulting in slower biological activity, clayey soil promotes microorganisms in the NSZD process to a greater extent because of clay minerals and humic matter providing favorable conditions for bacterial growth [50]. More precise results may be obtained through the addition of genetic quantification.

The effective gas diffusion coefficient of soil gases (O_2_, CH_4_, CO_2_, etc.) in the subsurface is a key parameter evaluated using the gas concentration gradient method. In the evaluation, accurate measurement or estimation of the effective diffusion coefficient will make the assessment results more accurate. If the parameters are estimated, accurate determination of soil porosity and moisture content is crucial. Additionally, although the positions of each sampling location were unknown before measurement, based on the data from this study, measuring gas concentrations in the lower portion of the oxidation zone and using them for evaluation calculations would underestimate the NSZD rate. If the measurements are taken in shallow near-surface locations, it would overestimate the NSZD rate. The appropriate position is located in the upper part of the oxidation zone [43].

The application hypothesis of the gas concentration gradient method is relatively simple and does not require a detailed description of the structure or properties of the contaminant, nor the determination or quantification of individual loss mechanisms to assess the overall mass loss rate. The required data are relatively easy to obtain, and the analysis is not overly complex. The current assessment of source zone mass loss rates is based on data obtained at specific times and locations, which can represent the current NSZD condition of the site through a single measurement [51]. However, whether and how the NSZD of the site will change in the future should be validated by multiple monitoring over a long period.

## 5. Conclusions

In this study, based on the soil gas concentration gradient method, the concentrations of a variety of soil gases (O_2_, CO_2_, CH_4_, VOCs, etc.) were detected, and the NSZD rate of a LNAPL-contaminated clayey soil site in China was evaluated. Combined with metagenomic sequencing technology, the key functional gene of NSZD, mcrA, was analyzed. The main attenuation processes at the site are methanogenesis and methane oxidation. Different from other sandy soil sites, there is a narrower methane oxidation zone in this clayey soil site, and the position in the vadose zone is shallower. The attenuation rate of the site, determined by O_2_ flux, ranged from 0.23 kg·m^−2^·a^−1^ to 1.15 kg·m^−2^·a^−1^, which falls within the moderate range compared to most previous studies. Although clayey soil is not good for gas transport, it may greatly promote microbial action. It is hard to say which of the two contributes more to the current NSZD rate. At present, we can only confirm the microbial action in the process of NSZD, but the specific mechanism of action needs to be further studied in the future. If each layer of soil is sampled for genetic quantitative testing, that will make the study results more accurate and detailed. Taken together, this study provides a method and data reference for assessing the attenuation rate of this site or other clayey soil sites in China, enriching the attenuation rate data of contaminated sites, and providing a realistic case for LNAPL attenuation scenarios in the clay formation.

## Figures and Tables

**Table 1:** Gas concentration gradient calculation.

Point	Depth (m)	O_2_ (kg/L/m)	CO_2_ (kg/L/m)	CH_4_ (kg/L/m)	VOCs (kg/L/m)
S1	0–0.7	−4.29 × 10^−5^	5.30 × 10^−5^	0	2.65 × 10^−8^
S2	0–0.3	−7.00 × 10^−5^	4.13 × 10^−5^	0	5.22 × 10^−8^
S3	0–0.3	−1.16 × 10^−5^	4.70 × 10^−5^	0.5	1.53 × 10^−8^
S4	0–0.4	−2.67 × 10^−5^	2.13 × 10^−5^	0	6.60 × 10^−8^

**Table 2:** Assessment of NSZD rate.

Point	S1	S2	S3	S4
Calculated Rate (kg·m^−2^·a^−1^)	0.04	0.71	1.19	0.27
Correction for Calculated Rate (kg·m^−2^·a^−1^)	-	0.67	1.15	0.23
Calculated Rate Using CO_2_ Flux (kg·m^−2^·a^−1^)	0.04	0.33	0.37	0.17
Correction for Calculated Rate Using CO_2_ Flux (kg·m^−2^·a^−1^)	-	0.29	0.33	0.13

The correction for calculated rate of background point S1 is not required. It is represented by “-”.

## Data Availability

The data presented in this study are available on request from the corresponding author.

## Supplementary Materials

The following supporting information can be downloaded at: https://www.mdpi.com/article/10.3390/life14030337/s1. Figure S1. The concentration of methane gas in groundwater.
