# Supplementary figures and images for: Assessing the Natural Source Zone Depletion of a Petroleum-Contaminated Clayey Soil Site in Southern China Combining Concentration Gradient Method and Metagenomics
Source: Life (Basel). 2024 Mar 4;14(3):337. doi: 10.3390/life14030337 (PMC10971796; doi:10.3390/life14030337)

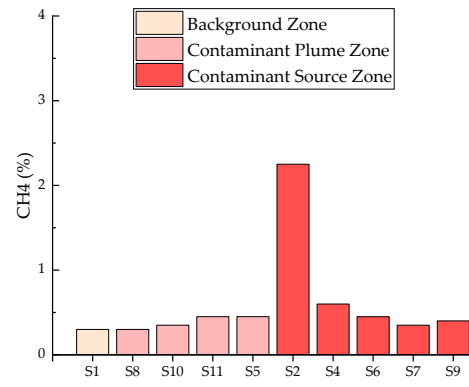

**Figure S1.** The concentration of methane gas in groundwater.

Supplement: Supplementary file 1 [file life-14-00337-s001.zip › life-2866147-supplementary.pdf]
